# Sense of Coherence and Physical Health. A Cross-Sectional Study Using a New Scale (SOC II)

**DOI:** 10.1100/tsw.2006.350

**Published:** 2006-10-09

**Authors:** Trine Flensborg-Madsen, Søren Ventegodt, Joav Merrick

**Affiliations:** ^1^ Quality of Life Research Center, Teglgårdstræde 4-8, DK-1452 Copenhagen K, Denmark; ^2^ Research Clinic for Holistic Medicine, Copenhagen, Denmark; ^3^ Nordic School of Holistic Medicine, Copenhagen, Denmark; ^4^ Scandinavian Foundation for Holistic Medicine, Sanvika, Norway; ^5^ Interuniversity College, Graz, Austria; ^6^ National Institute of Child Health and Human Development, Ministry of Social Affairs, Jerusalem, Israel; ^7^ Office of the Medical Director, Division for Mental Retardation, Ministry of Social Affairs, Jerusalem, Israel

**Keywords:** Antonovsky, sense of coherence scale, SOC II, predictability, physical health, comprehensibility, manageability, meaningfulness, Denmark

## Abstract

In this study, we constructed a new sense of coherence scale (SOC II), where we eliminated the notion of predictability (that life is meant to be predictable), which was present in the original SOC scale developed by Aaron Antonovsky (1923—1994) (SOC-29 and SOC-13). Our hypothesis was that SOC II would show a higher degree of association with physical health than the original SOC scale. In order to test this idea, we used a cross-sectional study including 4,648 Danes and used the three different health measures: self-evaluated physical health, physical symptoms, and self-evaluated psychological health. We found that SOC II was positively associated with all three health measures with the correlation coefficients 0.338, 0.282, and 0.578, respectively. Furthermore, we found dose response tendencies for all three health measures across groups of SOC, since health improved with a higher SOC. By means of regression analysis, we found that SOC was significantly associated with all three health measures after stratifying for demographic variables, life style variables, life form variables, and attitude variables, respectively. We conclude from this study that the SOC II scale we developed seems better associated with physical health than found with the original SOC scale. We also postulate that the concept of predictability was irrelevant, or even disturbing, and should not be included in the SOC scale.

## INTRODUCTION

A review of the original sense of coherence (SOC) scale showed that it was highly associated with psychological measures, but unable to show a strong association between SOC and physical health, as the inventor Aaron Antonovsky (1923–1994) claimed it would[1]. The SOC scale could only serve as a predictor for health measured by incorporating psychological aspects, while it was not able to explain health measured only by means of physical terms[1]. This is in strong contrast to the general belief of the importance of Antonovsky's idea of a strong association between SOC and physical health.

In a previous paper, we analyzed the SOC scale developed by Antonovsky (SOC-29 and SOC-13)[2] and, in a sequent study, we proposed some other questions for a new scale, the SOC II[3] in order to improve the original SOC scale. The questions chosen for SOC II were based on the same theory as the original scale developed by Antonovsky, but our operationalization is different[3].

The aim of this study was to construct the scale, SOC II, from the questions chosen in a previous paper[3] and to test whether SOC II is better associated with physical health than the original SOC scale. In order to test this hypothesis, we used a cross-sectional study with 4,648 Danes.

## METHODS

The study was carried out within the framework of the Copenhagen Perinatal Birth Cohort 1959–61 based on a follow-up study of this cohort[4]. The cohort consisted of 9,006 mothers and their 8,820 surviving children (from a total of 9,125 children) born at the University Hospital in Copenhagen (Rigshospitalet) during the 1959–1961 period. Using the CPR (Central Person Register) register, we succeeded in tracing 7,222 of the original children from the cohort, and this study consists of answers from the 4,648 persons who returned the questionnaire. The age of the participants at the time of the follow-up study was 31–33 years[4].

### Questionnaire

In a previous paper[3], we developed the questions that should be included in the new SOC scale, SOC II. The 35 questions were chosen among questions from the Self-Evaluated Quality of Life (SEQOL) questionnaire, which consisted of 317 items. In addition to a number of other things, the SEQOL measures the global quality of life and it is a self-administered questionnaire with items rated on different scales, from binary up to six-point and self-written scales[5,6,7].

The questions chosen to be included in SOC II were selected according to the framework presented in Table 1, which illustrates the theory of Antonovsky as described in a previous paper[3]. We included 11 questions relating to comprehensibility; 13 questions relating to manageability, and 11 questions relating to meaningfulness. Examples of some of the questions that were chosen from SEQOL are shown in Fig. 1.

**TABLE 1**
**Overview of the Frame from which the 35 Questions were Chosen**

| | Comprehensibility | Manageability | Meaningfulness |
|---|---|---|---|
| Self | 4 questions | 3 questions | 5 questions |
| Life | 3 questions | 4 questions | 3 questions |
| External world | 4 questions | 6 questions | 3 questions |

***"Do you see problems as a challenge?"*** **(Manageability/external world)**

1. Yes
2. Not sure
3. No

***"How satisfied are you with your life now?"*** **(Meaningfulness/life)**

1. Very satisfied
2. Satisfied
3. Neither satisfied nor dissatisfied
4. Dissatisfied
5. Very dissatisfied

**FIGURE 1.** Examples of two questions that were chosen from the SEQOL questionnaire to be included in SOC II.

## Measures

In order to describe health, we used three different health measures: physical health, physical health symptoms, and psychological health. The analyses were carried out using logistic regression analysis according to four different categories of possible confounders, respectively, demographic variables, life style variables, life form variables, and attitude variables (see Fig. 2). The confounders were divided into these groups with the intention of stretching from physical factors (demographic variables) towards consciousness (attitude variables). All measures used in the analyses are described below.

A score for *comprehensibility* was found in the following way:

((Total score of "self"/4) + (Total score of "life"/3) + (Total score of "external world"/4))/3

**FIGURE 2.** Calculation of the total score of comprehensibility.

- **Physical health** was measured by the question: "In relation to your physical health, how do you feel now?" (1) Very well, (2) well, (3) neither well or ill, (4) ill, (5) very ill.
- **Physical health symptoms** were measured by the question: "Do you have any of the following health symptoms now?" (1) No; (2) Yes somewhat; (3) Yes a lot; with the list including the 10 symptoms: pain or discomfort in (1) shoulder or neck; (2) back or buttocks; (3) arms, hands, legs, knee, hips, or joints; (4) headache; (5) rapid heart beat; (6) stomach pain; (7) indigestion, diarrhea, or constipation; (8) eczema, rash, or itching; (9) cold, head cold, or cough; (10) difficulty in breathing or breathlessness. Hence a physical health scale was constructed in which it was possible to score between 10 and 30 points. All items in the scale were positively correlated and Cronbach's alpha was 0.587
- **Psychological health** was measured according to the question: "In relation to your mental health, how do you feel now?" (1) Very well, (2) well, (3) neither well or ill, (4) ill, (5) very ill.

### *Model 1 (Demographic Variables)*

- **Gender**
- **Age** (annual)
- **Residence** — (1)Copenhagen, (2)in a large town or suburb of Copenhagen, (3)in a village, (4)in the country

### *Model 2 (Life Style Variables)*

- **Income group** — Own income plus the income of partner, if any; the income was split into 14 groups ranging from 6,667 EURO to over 266,667 EURO.
- **Body mass index (BMI)** — Calculated as weight (kg) per height squared (m).
- **Exercise** — "How many hours of exhausting exercise do you get a week?" (1) 0 h, (2) 30 min, (3) 1 h, (4) 2 h, (5) 3–7 h, (6) 8 h or more.
- **Alcohol intake** — "How much alcohol did you drink in the last complete weekday?" plus: "How much alcohol did you drink in total last weekend?"; measured in drinks.
- **Tobacco consumption** — "Do you smoke?" (1) Yes, daily; (2) yes, now and then; (3) no, I stopped less than a year ago; (4) no, I stopped more than a year ago; (5) no, I have never smoked.
- **Vegetarian** — "I am a vegetarian" (1) No; (2) yes.

### *Model 3 (Life Form Variables)*

- **Marital status** — (1) Single, (2) living with partner, (3) married.
- **Social network I (quantitatively)** — "How many close friends or family members do you have daily contact with (for example, see them or talk to them on the telephone?" (1) None, (2) one a day, (3) two a day, (4) three a day, (5) four or more.
- **Social network II(qualitatively)** — "Do you have a friend with whom you can discuss any subject?" (1) Yes, (2) not sure, (3) no.
- **Sexual orientation** — (1) Heterosexual, (2) bisexual, (3) homosexual.
- **Satisfaction with sex life** — "How satisfied are you with your sex life now?" (1) Very satisfied, (2) satisfied, (3) neither satisfied nor dissatisfied, (4) dissatisfied, (5) very dissatisfied.
- **Social work environment** — "How good is your work environment?" (1) Very good, (2) good, (3) neither good nor poor, (4) poor, (5) very poor.

### *Model 4 (Attitude Variables)*

- **Quality of life —** "How would you assess the quality of your life?" (1) Very high, (2) high, (3) neither high nor low, (4) low, (5) very low.
- **Do you often worry?** — (1) Yes, (2) not sure, (3) no.
- **Do you think that other people speak well of you?** — (1) Yes, (2) not sure, (3) no.
- **Do you feel you are loved?** — (1) Yes, (2) not sure, (3) no.

## Construction of the SOC Scale

The 35 questions chosen for the SOC scale were all answered on either a three-point scale or a five-point scale. Therefore, the five-point scales were recoded into three-point scales by lumping together the two top alternatives and also the two bottom alternatives. Furthermore, all questions were coded so that the most favorable answer gave one point, while the least favorable answer gave three points.

The total scores of each concept (*comprehensibility*, *manageability,* and *meaningfulness)* were found for each person using the procedure shown in Fig. 2 for *comprehensibility*. Hence it was possible to score between one and three points in each domain (each box in Table 1), and between three and nine points in each concept (each column in Table 1). The mean scores of the concepts of comprehensibility, manageability, and meaningfulness are presented in Table 2 with different coding of the missing variables.

**TABLE 2**
**Mean Scores of Comprehensibility, Manageability, Meaningfulness, and SOC II**

| | Comprehensibility | Manageability | Meaningfulness | SOC II |
|---|---|---|---|---|
| Mean score (individuals with one or more missing value excluded) | 4.22 | 4.24 | 4.28 | 12.44 |
| Mean score (missing values = 1) | 4.24 | 4.24 | 4.38 | 12.86 |
| Mean score (missing values = 3) | 4.51 | 4.35 | 4.56 | 13.42 |

The results in Table 2 revealed that the mean scores of the three dimension's comprehensibility, manageability, and meaningfulness were very nearly independent of the coding of the missing variables. However, the mean scores were slightly lower for the individuals with one or more missing value excluded than they were in the other two situations. This showed us that the persons with missing values in general had a lower SOC than those who answered all the questions. The total SOC II score for each person was calculated by adding together the points from each of the three concepts; hence, the SOC II scale is ranging from nine to 27 points. The mean SOC II score was 12.44, when the missing values were excluded, 12.86 when the missing values were coded as one, and 13.42 when the missing values were coded as three.

By use of a sensitivity analysis, we discovered that the correlation coefficient between the SOC II scale and the three health measures were very similar regardless of the coding of the missing values in the SOC scale II (Table 3). This illustrates that the missing values were randomly distributed according to the association between SOC II scale and the health measures that we used.

**TABLE 3**
**Sensitivity Analysis: Person Correlations Between SOC II (with Different Coding of the Missing Variables) and Health (all $p < 0.0005$)**

| | Physical Health | Physical Symptoms | Psychological Health |
|---|---|---|---|
| SOC II (without missing values) | 0.338 | 0.282 | 0.578 |
| SOC II (missing values = 1) | 0.369 | 0.279 | 0.616 |
| SOC II (missing values =3) | 0.361 | 0.264 | 0.598 |

Since we did not want to create bias in relation to the coding of missing values, we decided to delete the subjects with missing values in the variables used in the analyses of SOC II vs. health, etc.; hence, we used the so-called complete-subject analysis. The method is valid whenever subjects with complete data are a random sample of all the subjects in the study, and are random samples within levels of variables that are used for stratification[8]. Based on the sensitivity analysis of the correlations between SOC and the three health measures, we found that it was reasonable to delete the subjects. Therefore, our results are presented as to a situation where individuals with missing values were excluded. Cronbach's alpha of the SOC II scale was found to be 0.862 when the missing values were excluded, which points to a high degree of parallelism in the subjects' response patterns.

## Statistical Analyses

Baseline characteristics of the population were computed for different groups of SOC, see Table 4. The subjects were divided into five groups by the SOC II total score according to the actual range. The subjects

**TABLE 4**
**Characteristics of Subjects Divided by Category of SOC**

| | Group I (N = 1,589) Mean (SD) | Group II (N = 821) Mean (SD) | Group III (N = 198) Mean (SD) | Group IV (N = 10) Mean (SD) |
|---|---|---|---|---|
| Physical health (1 = very well and 5 = very ill) | 1.68 (0.72) | 2.02 (0.82) | 2.44 (0.83) | 3.40 (0.84) |
| Physical symptoms (10 = no symptoms and 30 = all symptoms felt a lot) | 12.51 (2.10) | 13.27 (2.42) | 14.53 (2.81) | 16.44 (4.36) |
| Psychological health (1 = very well and 5 = very ill) | 1.51 (0.63) | 2.11 (0.83) | 2.97 (0.91) | 3.40 (0.97) |
| **Demographic variables:** | | | | |
| Sex (1 = woman and 2 = man) | 1.49 | 1.44 | 1.43 | 1.40 |
| Age (years) | 31.89 (0.73) | 31.90 (0.73) | 31.95 (0.69) | 32.00 (0.67) |
| Residence (1 = Copenhagen and 4 = in the country) | 1.90 (0.88) | 1.90 (0.86) | 1.88 (0.82) | 1.80 (0.79) |
| **Life style variables:** | | | | |
| Income groups (1 = 6667 EURO, 14 = over 266.667 EURO) | 7.06 (1.67) | 6.80 (1.61) | 6.62 (1.77) | 6.11 (1.90) |
| Exercise (1 = 0 h and 5 = 4 h or more) | 3.51 (1.40) | 3.14 (1.48) | 3.15 (1.55) | 3.30 (1.42) |
| Alcohol in units | 6.24 (6.82) | 6.24 (7.20) | 7.44 (8.70) | 7.40 (10.80) |
| Tobacco consumption (1 = yes and 5 = no, never did) | 2.90 (1.79) | 2.81 (1.80) | 2.77 (1.76) | 3.20 (1.69) |
| BMI kg/($m^2$) | 23.55 (3.46) | 23.29 (3.70) | 23.19 (3.63) | 21.88 (3.25) |
| Vegetarian (1 = no and 2 = yes) | 1.21 | 1.18 | 1.30 | 1.00 |
| **Life form variables:** | | | | |
| Marital status (1 = single and 3 = married) | 2.57 (0.60) | 2.49 (0.61) | 2.41 (0.64) | 2.40 (0.70) |
| Social network I (1 = none and 5 = 4 or more) | 3.60 (1.30) | 3.19 (1.36) | 2.75 (1.29) | 3.10 (1.79) |
| Social network II (1 = close friend and 3 = no close friend) | 1.28 (0.61) | 1.52 (0.75) | 1.79 (0.86) | 1.40 (0.70) |
| Sexual orientation (1 = heterosexual and 3 = homosexual) | 1.03 (0.25) | 1.02 (0.19) | 1.03 (0.23) | 1.00 (0.00) |
| Sexual satisfaction(1 = very satisfied and 3 = very dissatisfied) | 1.87 (0.84) | 2.46 (1.04) | 3.06 (1.09) | 3.30 (0.95) |
| Work environment (1 = very good and 5 = very poor) | 1.78 (0.77) | 2.22 (0.94) | 2.68 (1.04) | 3.60 (1.07) |
| **Attitude variables:** | | | | |
| Quality of life (1 = very high and 5 = very low) | 1.59 (0.56) | 2.18 (0.67) | 2.97 (0.78) | 3.60 (0.70) |
| Often worry (1 = yes and 3 = no) | 2.39 (0.78) | 1.90 (0.85) | 1.37 (0.66) | 1.20 (0.42) |
| Other people speak well (1 = yes and 3 = no) | 1.28 (0.54) | 1.48 (0.62) | 1.66 (0.67) | 2.00 (0.67) |
| Feel loved (1 = yes and 3 = no) | 1.05 (0.22) | 1.14 (0.36) | 1.42 (0.60) | 1.50 (1.07) |

with the strongest SOC II formed group I, and the subjects with the weakest SOC II formed group V. Because of the skew distribution of the SOC II total score, SOC V, which would include very weak SOC individuals, contained no individuals. Furthermore, group IV was small in size with only 10 individuals. The SOC groups formed are as follows:

| | | |
|---|---|---|
| SOC group I: | 9–12.6 points | n = 1,589 (60.6%) |
| SOC group II: | 12.6–16.2 points | n = 821 (31.4%) |
| SOC group III: | 16.2–19.8 points | n = 198 (7.6%) |
| SOC group IV: | 19.8–23.4 points | n = 10 (0.4%) |
| SOC group V: | 23.4–27.0 points | n = 0 (0%) |

Linear regression was carried out using the four different categories of possible confounders, respectively. Each of the models was applied using self-evaluated physical health, physical symptoms, and self-evaluated psychological health, respectively, as dependent variables (Tables 5–8).

**TABLE 5**
**Model 1 (Demographic Variables), Linear Regression Analysis with Health as the Dependent Variable**

| | Physical Health | | | Physical Symptoms | | | Psychological Health | | |
|---|---|---|---|---|---|---|---|---|---|
| | β | S.E. | Sig | β | S.E. | Sig | β | S.E. | Sig |
| SOC II | 0.119 | 0.007 | * | 0.284 | 0.020 | * | 0.217 | 0.006 | * |
| Sex | –0.001 | 0.030 | NS | –0.582 | 0.091 | * | –0.012 | 0.028 | NS |
| Age (annual) | –0.027 | 0.021 | NS | 0.022 | 0.062 | NS | –0.022 | 0.019 | NS |
| Residence | 0.019 | 0.017 | NS | –0.020 | 0.052 | NS | –0.014 | 0.016 | NS |

* $p < 0.0001$; NS = Nonsignificant ($p > 0.05$).

**TABLE 6**
**Model 2 (Life Style Variables), Linear Regression Analysis with Health as the Dependent Variable**

| | Physical Health | | | Physical Symptoms | | | Psychological Health | | |
|---|---|---|---|---|---|---|---|---|---|
| | β | S.E. | Sig | β | S.E. | Sig | β | S.E. | Sig |
| SOC II | 0.118 | 0.007 | ** | 0.294 | 0.020 | ** | 0.217 | 0.006 | ** |
| Income | –0.013 | 0.009 | NS | –0.075 | 0.028 | * | –0.003 | 0.009 | NS |
| Exercise | –0.057 | 0.010 | ** | –0.051 | 0.031 | NS | –0.008 | 0.010 | NS |
| Alcohol | 0.000 | 0.002 | NS | –0.028 | 0.006 | ** | 0.004 | 0.002 | NS |
| Tobacco | –0.008 | 0.008 | NS | –0.147 | 0.026 | ** | –0.005 | 0.008 | NS |
| BMI | 0.022 | 0.004 | ** | 0.053 | 0.013 | ** | 0.001 | 0.004 | NS |
| Vegetarian | –0.012 | 0.014 | NS | –0.005 | 0.041 | NS | 0.007 | 0.013 | NS |

* $p < 0.05$; ** $p < 0.0001$; NS = Nonsignificant ($p > 0.05$).

## RESULTS

Table 4 shows that all three health measures increased with a higher SOC score, which means that the health problems increase when SOC gets poorer. The majority of the variables show dose-response tendencies according to SOC group; especially for the attitude variables, the dose-response tendencies were very clear: The group with the best SOC had a better quality of life, seldom worried, were more likely to believe that other people spoke well of them and felt more loved than the group with the worst SOC. Other characteristics of the people in the best SOC group were persons with a higher income, higher BMI, more likely to be married, more satisfied with their sex life, and more satisfied with their work life.

Table 5 shows that a positive significant association existed between SOC II and all three health measures when stratifying for demographic variables. The positive beta values revealed that a high SOC score (which means a low SOC) was associated with a high health score (which means bad health). The significant negative beta value of sex (gender), in relation to the physical symptoms, implied that women had more physical symptoms than men.

**TABLE 7**
**Model 3 (Life Form Variables), Linear Regression Analysis with Health as the Dependent Variable**

| | Physical Health | | | Physical Symptoms | | | Psychological Health | | |
|---|---|---|---|---|---|---|---|---|---|
| | β | S.E. | Sig | β | S.E. | Sig | β | S.E. | Sig |
| SOC II | 0.108 | 0.008 | ** | 0.282 | 0.025 | ** | 0.191 | 0.008 | ** |
| Marital status | 0.021 | 0.026 | NS | 0.050 | 0.079 | NS | –0.052 | 0.024 | * |
| Social network I | 0.010 | 0.012 | NS | 0.023 | 0.036 | NS | 0.003 | 0.011 | NS |
| Social network II | –0.010 | 0.023 | NS | –0.025 | 0.071 | NS | 0.036 | 0.021 | NS |
| Sexual orientation | 0.047 | 0.070 | NS | 0.386 | 0.207 | NS | 0.162 | 0.065 | * |
| Sexual satisfaction | 0.026 | 0.017 | NS | –0.030 | 0.053 | NS | 0.069 | 0.016 | ** |
| Work environment | 0.064 | 0.019 | * | 0.105 | 0.056 | NS | 0.053 | 0.017 | ** |

* $p < 0.05$; ** $p < 0.0001$; NS = Nonsignificant ($p > 0.05$).

**TABLE 8**
**Model 4 (Attitude Variables), Linear Regression Analysis with Health as the Dependent Variable**

| | Physical Health | | | Physical Symptoms | | | Psychological Health | | |
|---|---|---|---|---|---|---|---|---|---|
| | β | S.E. | Sig | β | S.E. | Sig | β | S.E. | Sig |
| SOC II | 0.070 | 0.004 | ** | 0.140 | 0.028 | ** | 0.111 | 0.008 | ** |
| Quality of life | 0.185 | 0.025 | ** | 0.259 | 0.077 | * | 0.366 | 0.022 | ** |
| Worry | –0.050 | 0.019 | * | –0.465 | 0.059 | ** | –0.140 | 0.017 | ** |
| How other people speak | 0.035 | 0.026 | NS | 0.261 | 0.079 | * | –0.008 | 0.022 | NS |
| Feel loved | 0.015 | 0.047 | NS | 0.107 | 0.146 | NS | 0.139 | 0.042 | * |

* $p < 0.05$; ** $p < 0.0001$; NS = Nonsignificant ($p > 0.05$).

Table 6 shows that a positive significant association existed between SOC and all three health measures when stratifying for life style variables. The *p*-values revealed that there were different informative life style features, depending on the health measure. In each line, the regression coefficients generally had the same sign in all three columns, which is the sign that agrees with common knowledge, meaning that bad health is associated with low income, low exercise, daily smoking, and a high BMI. Alcohol seemed only to be associated with physical symptoms, thus many symptoms were associated with low alcohol consumption (this should not be taken as pure fact, since it is known that the relationship between health and alcohol consumption is not linear, but j-shaped or u-shaped[9,10]). Being a vegetarian did not seem to have relation to any of the health measures.

Table 7 shows that SOC was associated positively with all three health measures when stratifying for the life form variables. None of the life form variables were significantly associated with physical health or physical symptoms in the regression analysis. However, being single and unsatisfied with your sex life seemed to be bad for psychological health.

Table 8 shows that the attitude variables were the most important factors according to the association between SOC II and the three different health measures, since the regression coefficients were approximately halved compared to the coefficients seen in Tables 5–7. Hence, the regression coefficients for physical health were found to be 0.119, 0.118, and 0.117, respectively, in models 1, 2, and 3, whereas it was 0.070 in model 4 when stratifying for attitude variables. The regression coefficients for physical symptoms were 0.284, 0.294, and 0.296, respectively, in models 1, 2, and 3, whereas it was 0.140 in model 4. The same pattern was found for psychological health.

The most important factors for health were the quality of life and whether you often worried, since they were both significantly associated with all three health measures. The regression coefficients showed that a low quality of life and worry often was associated with bad health. In addition, a large number of physical symptoms was associated with that person's interpretation of whether other people spoke well of you, and bad psychological health was associated with the fact of not feeling loved.

## DISCUSSION

### Methodological Issues

The technique of sampling and data collection, which took place several years before these analyses, is believed to be applicable to the aim of the study. The design is weak as it is a cross-sectional study and, hence, no causal conclusions can be drawn and further longitudinal studies are needed to confirm the direction of the associations that we found. Except for the age of the respondents, the sample is more or less population representative, as it comprises people who were born at the same hospital in a given period. The sample was of a very large size and was equal in size between genders. Cronbach's alpha coefficient for SOC II was 0.862, which points to a high degree of parallelism in the subjects' response patterns. However, Cronbach alpha does not inform whether the scale is theoretically meaningful. The results of this study are built on results from a previous study in which we chose the questions that should be included in SOC II; for further information of this study, we refer to two other papers[2,3].

### Result Issues

In the current cross-sectional study of 4,648 Danes, our newly developed sense of coherence scale, SOC II, seemed to be correlated significantly with the three health measures: physical health, physical symptoms, and psychological health. The relation between SOC and health has been well established, hence the original SOC scale developed by Antonovsky showed to be highly associated with psychological measures[11,12,13,14,15,16,17,18,19,20,21], including stress[22,23,24,25,26,27,28] and behavioral aspects[29,30,31,32,33,34]. However, the results have been diverse related to physical health[35,36,37,38,39,40,41,42]. The original SOC scale seemed only to serve as a predictor for health that was measured by incorporating psychological aspects, while it was not capable of explaining health elicited only by means of physical terms[1].

In the present study, we found moderate correlation coefficients between SOC II and health: 0.338 for physical health, 0.282 for physical symptoms, and 0.578 for psychological health. Despite the fact that the Pearson correlation coefficient was quite a rough measure that did not give weight to any confounders, our results nevertheless were better than the majority of the results that we were able to trace in a review of more than 50 papers concerned with SOC and physical health[1]. In the review, we concluded that several studies found the association between SOC and physical health to be nonsignificant, and that the other studies generally found the correlation coefficient to be between 0.17–0.51, with the highest correlations found in studies that divided the study population into minor groups in order to find a higher correlation[1].

In addition to our correlation coefficients, we found dose-response tendencies according to the health measures in different SOC groups due to the fact that health improved with a better SOC. Furthermore, our regression analysis revealed that the association between SOC and all three health measures stayed significant when stratifying for demography, life style, and life form variables, with regression coefficients ranging from 0.117–0.296. Since the regression coefficients in a model that did not stratify for any variables were 0.120, 0.294, and 0.216, respectively, for the three health measures, it can be argued that the demography, life style, and life form variables did not have any noteworthy influence on the relation between SOC and health. The only demographic variable important for health was gender, since women seemed to have more physical symptoms than men. The life style variables: income, exercise, smoking, and BMI were all associated to either physical health, physical symptoms, or both, in agreement with common knowledge, so that low income, low exercise, daily smoking, and a high BMI was associated with bad physical health. None of the life form variables were associated with physical health.

Contrarily, the attitude variables turned out to have a rather big influence, since the regression coefficients altered to be half the size when stratifying for attitude variables being 0.070, 0.140, and 0.111, respectively. The attitude variables quality of life and worrying were associated with all three health measures. As illustrated in Fig. 2, the groups of possible confounders were chosen with the intention of stretching from physical factors (demographic variables) towards consciousness (attitude variables). In our study, the physical factors, such as demography, life style, and life form, did not have any noteworthy influence. Instead, it was the conscious variables that seemed important.

The overall picture of our analyses signified the fact that SOC II was better associated with physical health than the original scale developed by Aaron Antonovsky. In our new SOC scale, we eliminated the notion of life's predictability and we conclude that the concept of predictability is irrelevant, and even disturbing, to include in a SOC scale. However, we did not deal with the other problem that we believe exists in the scale, i.e., that the SOC scale mixes emotional aspects into an otherwise mental construct. In other papers, we will show that the distinction between mentality and emotionality is very essential when it comes to the association to health[43,44], and we found an even higher correlation between SOC and physical health by using the measure "emotional sense of coherence" (SOC-E)[44].

## CONCLUSION

In the present study, we developed a new sense of coherence scale, SOC II, based on the selection of questions in a previous paper[3], and we investigated the association between SOC II and three different health measures: self-evaluated physical health, physical symptoms, and self-evaluated psychological health. Our hypothesis was that the new SOC II scale would demonstrate a higher association with physical health than what has previously been found according to the original SOC scale developed by Antonovsky. The reason for this hypothesis was that we eliminated the notion of life's predictability, which was present in Antonovsky's original scale.

Our hypothesis was confirmed when we found moderately significant correlation coefficients between SOC II and all three health measures. The coefficients were higher than what has been found in most other studies concerned with the association between SOC and health. In addition, we carried out linear regression analysis in which we stratified for a number of variables, and even that could not alter the considerable association between SOC II scale and the three health measures. We therefore conclude from this study that modifications should be made to the original SOC scale developed by Antonovsky in order to reveal the mystery of physical health, and the notion of predictability should be eliminated from the scale. In this paper, we presented a suggestion for such a scale, however, the SOC II ought to be tested in other studies in order to verify its use.
